# Artificial Intelligence‐Enabled Electrocardiogram for the Detection and Management of Cancer Therapy‐Related Cardiotoxicity

**DOI:** 10.1002/cai2.70042

**Published:** 2025-12-28

**Authors:** Wenhua Song, Runze Gao, Tong Liu

**Affiliations:** ^1^ Tianjin Key Laboratory of Ionic‐Molecular Function of Cardiovascular Disease, Department of Cardiology Tianjin Institute of Cardiology The Second Hospital of Tianjin Medical University Tianjin China

**Keywords:** artificial intelligence, cardiotoxicity, electrocardiogram

**Abbreviations**
AF: atrial fibrillation
AI: artificial intelligence
AI‐ECG: AI‐enhanced electrocardiography
AUC: area under the operating characteristics curve
CNN: convolutional neural networks
CTRC: cancer therapy‐related cardiotoxicity
CTRCD: cancer therapy‐related cardiac dysfunction
DL: deep learning
ECG: electrocardiogram
HF: heart failure
ICIs: immune checkpoint inhibitors
LVD: left ventricular dysfunction
ML: machine learning

## 1 Introduction

Cancer remains a predominant cause of morbidity and mortality in contemporary society globally. Whilst therapeutic advancements such as chemotherapy, radiotherapy, immunotherapy, have substantially improved survival outcomes in cancer patients, these interventions are frequently associated with cardiotoxic effects [1, 2, 3]. Notably, the incidence of cardiotoxicity among cancer patients exhibits substantial heterogeneity across studies, with documented rates between 3.8% and 37.5% [4].

Guidelines from learned cardiology societies recommend the use of baseline electrocardiograms (ECGs) for all patients prior to the initiation of cancer therapy [1]. Thus, ECG data should be readily available as part of the routinely collected health data. Artificial intelligence (AI) can integrate multimodal data (e.g., imaging, pathology, and clinical records) together with ECG analysis to optimize diagnostic accuracy and treatment personalization [5].

Feng et al. recently published a review article in *Cancer Innovation* on the use of AI for breast cancer management. The authors highlighted the significance of constructing patient‐oriented AI algorithms in management of breast cancer patients [6]. AI has been extensively employed across all facets of breast cancer management, including diagnosis, risk prediction, treatment response evaluation, and prognosis assessment. As the most widely accessible and standardized cardiac diagnostic tool, the integration of AI with ECG interpretation has the potential to transform cardiovascular care for cancer patients. Here, we evaluate the evidence for AI‐enabled ECG analysis in cancer therapy‐related cardiotoxicity (CTRC) detection and its emerging role in risk stratification for high‐risk oncology patients, also analyze the implications for clinical decision‐making in cardio‐oncology.

## 2 AI Applied to the ECG in Cardiology and Cardio‐Oncology

The integration of large‐scale ECGs with comprehensive data fields from clinical databases has enabled the development of AI models with high sensitivity to detect diverse cardiovascular pathologies [7, 8, 9]. Unlike conventional ECG interpretation that requires the manual interpretation by cardiologists, AI‐based algorithms demonstrate distinct advantages including operator independence, rapid processing capability, and integrated diagnostic‐prognostic functionality [10]. AI primarily encompasses two dominant approaches in contemporary healthcare applications, including machine learning and deep learning (DL). Notably, convolutional neural networks (CNN)—a specialized DL architecture—have become particularly transformative in medical image analysis due to their exceptional capability to deal with spatial hierarchies in imaging data [11].

Numerous studies have indeed demonstrated the effectiveness and potential clinical practicality of AI‐ECG in evaluating various cardiac conditions, which showed promising accuracy of different AI algorithms in the detection of left ventricular dysfunction (LVD), arrhythmias, myocardial hypertrophy, ischemic disease, cardiomyopathy, and pulmonary hypertension [12, 13]. CNN‐based AI‐ECG models exhibit robust performance in cardiovascular risk stratification for oncology patients. It was reported that area under the receiving characteristic operating (ROC) curve (AUC) for AI‐ECG model for predicting LVD was up to 0.93, and it can be used for risk stratification of LVD in patients with blood or solid tumors after anticancer treatment [8, 14, 15]. Meanwhile, the AI‐ECG model can also be used to predict the occurrence of arrhythmias represented by atrial fibrillation (AF) in cancer patients [7]. In summary, AI‐ECG has demonstrated significant clinical utility across both oncologic and general populations.

## 3 AI‐Enabled ECG in Detection and Prediction of CTRC

Several AI‐ECG models have recently been developed for the detection and prediction of CTRC. A cohort of 703 participants with breast cancer was analyzed by Jacobs et al., who demonstrated high diagnostic performance for LVD—their AI‐ECG model detected an EF < 50% and ≤ 35% after anthracycline therapy with an AUC of 0.93 and 0.94, respectively [14]. Another study from Japan evaluated the efficacy of AI‐ECG models for detecting anthracycline chemotherapy‐related cardiotoxicity. An AI‐ECG model based on CNN developed using data from 1011 patients was constructed, showing considerable ability to recognize LVD (HR: 2.66 for high risk) [15]. These studies thus support the use of AI‐ECG for LVD screening after anthracycline‐based chemotherapy.

Beyond conventional chemotherapy, immunotherapy has revolutionized contemporary oncology practice, with immune checkpoint inhibitors (ICIs) representing a pivotal therapeutic advancement. However, ICIs are associated with significant immune‐related adverse events, particularly cardiovascular complications. A recent multicenter study conducted by Chinese investigators systematically evaluated clinical characteristics and ECG parameters of 419 cancer patients receiving ICIs [16]. XGBoost models demonstrated the highest predictive performance with an AUC of 0.83, with variables selected based on each model's characteristics. Of concern, of the 10 features included in this model, five were extracted from the ECG. The model addressed the modern challenge of ICI‐related cardiotoxicity, providing crucial risk stratification to guide preventive measures and alternative treatment strategies.

Güntürkün et al. conducted a pioneering investigation into AI‐ECG applications for cardiotoxicity monitoring in childhood cancer survivors, which is significant for predicting long‐term cardiomyopathy, thereby enhancing risk stratification [17]. Their study focused on adult survivors of childhood malignancies who had received potentially cardiotoxic treatments (radiation and/or anthracycline‐based chemotherapy). The developed AI‐ECG model demonstrated superior predictive accuracy (AUC: 0.87) for LVD compared to conventional clinical markers (AUC: 0.69), which further confirmed that AI‐ECG model might be potential for the early identification of cardiomyopathy before symptom onset.

## 4 AI‐Enabled ECG in CTRC Risk Stratification and Management

Early diagnosis and treatment of heart failure (HF) increase the chance of complete left ventricular function recovery, highlighting the relevance of developing new techniques and protocols for early management of cancer therapy‐induced LVD [4, 18]. Oikonomou et al. have reported an AI‐ECG model that stratified the risk of cancer therapeutics‐related cardiac dysfunction (CTRCD), from a cohort of 3364 patients from five hospitals from the United States who received anthracyclines, trastuzumab, or ICI. Using AI‐ECG, left ventricular systolic dysfunction screening was associated with 2‐fold and 4.8‐fold increase in the incidence of the composite CTRCD endpoint (adj.HR: 2.22, 95% CI: 1.63–3.02), and LVEF < 40% (adj.HR: 4.76, 95% CI: 2.62–8.66), respectively [8].

Building upon these findings, AI‐ECG models demonstrate significant potential in detecting or predicting critical immunotherapy‐related cardiovascular outcomes. A multimodal fusion AI model combined ECG, demographics, comorbidities, and lab tests results was built to predict myocarditis and adverse cardiovascular events in cancer patients starting ICI therapy. The model exhibits good performance with AUC of 0.72 [19]. According to the 2022 ESC cardio‐oncology guidelines [1], a baseline ECG is recommended for all patients before cancer therapy (chemotherapy, immunotherapy, or radiotherapy), with surveillance frequency thereafter tailored to individual risk profiles. The application of AI algorithms to ECG analysis has emerged as a valuable tool for predicting and prognosticating various conditions in the general population, particularly immune‐related adverse events. These models show promise for not only the early detection of cardiac pathology but also risk stratification and implementation of preventative therapies in cancer patients. Patients with high risk of ICI‐associated myocarditis should use these agents with caution or switch to alternative treatment regimens. AI‐ECG algorithms have also shown promise in the early stratification of arrhythmias, with particularly regard to AF. Christopoulos et al. applied an AI‐ECG algorithm that targeted the prediction of AF, a less common focus compared to the prevalent LVD‐centric approaches from 754 newly diagnosed chronic lymphocytic leukemia patients, reporting a good risk stratification of AF (HR: 3.9; 95% CI: 2.6–5.7; *p* < 0.001) [7]. The AI‐ECG model provides important evidence for early risk stratification and individualized clinical management of CTRC.

To date, AI‐ECG has demonstrated the capability to identify treatment‐related toxicities across multiple tumor types [7, 8, 14, 15, 17, 19]. As reported in the review by Feng et al. [6], AI algorithms have served as an important auxiliary tool in the clinical management of breast cancer. This evidence further underscores the value of AI‐ECG in the detection and monitoring of breast CTRC. A major current focus involves the early detection of HF and risk stratification following anthracycline‐based chemotherapy, with model performance reaching an AUC of up to 0.93 [14]. The AI‐ECG model applied to a cohort including breast cancer patients receiving ICI showed promising utility in identifying immune‐related adverse events, such as ICI‐associated myocarditis [19]. Nonetheless, other forms of cardiotoxicity—including arrhythmias—in breast cancer patients undergoing anticancer treatments remain underexplored and warrant further investigation.

## 5 Limitations and Future Directions

Routine ECG assessment exhibits limited sensitivity for detecting certain types of CTRC, particularly those characterized by subtle structural, functional, or biochemical alterations that do not promptly produce detectable electrical abnormalities. These include: (1) subclinical LVD: while current AI‐ECG models show promising performance in identifying and predicting reduced LVEF, their ability to recognize HF with preserved ejection fraction remains uncertain; (2) microvascular dysfunction without overt ECG changes: studies have demonstrated that single‐lead ECG under stress conditions can significantly enhance the diagnostic accuracy of AI‐ECG models for ischemic heart disease [20]. However, the applicability and efficacy of this approach in the context of CTRC have not yet been established; (3) biomarker‐positive cardiotoxicity without electrical abnormalities: in cases where cardiotoxicity is only reflected by elevated biomarkers (such as troponin or brain natriuretic peptide), especially without systolic impairment, the ECG may often appear normal, limiting its utility as a standalone diagnostic tool.

Future AI‐ECG models can consider incorporating continuous ECG data captured by wearable devices, which would have the potential to provide real‐time detection and monitoring capabilities, and achieve dynamic risk assessment during cancer treatment. Building upon current technological foundations, next‐generation AI‐ECG models are poised to incorporate comprehensive clinical data fields beyond ECG features, while expanding their diagnostic scope beyond LVD, myocarditis, and arrhythmias to detect more subtle treatment‐related cardiac pathologies, such as myocardial fibrosis, subclinical HF, and coronary artery injury. These advanced systems may ultimately integrate with digital twin technology, enabling dynamic risk stratification for early prevention while simultaneously facilitating personalized long‐term treatment planning for patients with established CTRC (Table 1).

**Table 1:** Current research on AI‐ECG applications in cardio‐oncology.

References	Study cohort	Study region	Sample	Cancer	Anticancer treatment	AI‐model	Endpoints	Result
[17]	Güntürkün et al. (2021)	USA	1217	Pediatric tumors	Anthracyclines radiation	Extreme Gradient Boosting	Late‐onset cardiomyopathy (LVEF < 50% and/or LVEF drop ≥ 10%)	AUC: 0.87
[14]	Jacobs et al. (2024)	Belgium	703	Breast cancer	Anthracyclines	CNN	LVEF < 50% LVEF < 35%	AUC:0.93 AUC:0.94
[7]	Christopoulos et al. (2024)	USA	754 220	Chronic lymphocytic leukemia	Unspecified treatment Bruton tyrosine kinase inhibitors	CNN	Incident AF	HR: 3.9 for high AI risk patients HR: 2.8 for high AI risk patients
[15]	Yagi et al. (2024)	USA	1011	Blood and solid tumors	Anthracyclines	CNN	LVEF < 53% and/or LVEF drop > 10%	HR: 2.66 for high AI risk patients
[8]	Oikonomou et al. (2025)	Yale	1550	Breast cancer non‐Hodgkin lymphoma	Anthracyclines trastuzumab	CNN	Cardiomyopathy and/or HF and/or LVEF < 50% LVEF < 50% LVEF < 40%	HR: 3.55 for high AI risk patientsHR: 4.88 for high AI risk patients HR: 13.52 for high AI risk patients
[19]	Ayoub et al. (2025)	USA	2258	Lung/breast cancer, gastrointestinal cancer, melanoma, renal/genitourinary cancer, hepatobiliary cancer	ICI	A multimodal joint fusion AI model by combining 1‐D convolution and multilayer perceptron	ICI‐related myocarditis and major adverse cardiovascular events (transient ischemic attack/stroke, new diagnosis of heart failure, myocardial infarction, and cardiac death)	AUC:0.72
[16]	Li et al. (2025)	China	419	NA	ICI	Logistic regression Random forest eXtreme Gradient Boosting	Immunotherapy‐related cardiotoxicity within 30 days	AUC:0.80 AUC:0.74 AUC:0.83

Abbreviations: AUC, area under the operating characteristics curve; CNN, convolutional neural networks; HR, hazard ratio; ICI, immune checkpoint inhibitor; LVEF, left ventricular ejection fraction; NA, not available.

## 6 Conclusion

Despite significant advancements in AI‐ECG applications, considerable opportunities remain to enhance its utility in cardio‐oncology. AI‐ECG algorithms particularly promising for reducing the burden of cardiovascular sequelae in cancer survivors, ultimately improving long‐term outcomes.

## Author Contributions

**Wenhua Song:** writing – original draft (equal), investigation (equal). **Runze Gao:** investigation (equal), writing – original draft (equal). **Tong Liu:** conceptualization (lead), writing – review and editing (lead), funding acquisition (lead).

## Ethics Statement

The authors have nothing to report.

## Consent

The authors have nothing to report.

## Conflicts of Interest

Professor Tong Liu is the member of the *Cancer Innovation* Editorial Board. To minimize bias, he was excluded from all editorial decision‐making related to the acceptance of this article for publication. The remaining authors declare no conflicts of interest.

## Data Availability

The authors have nothing to report.
